# Effects of *Bacillus licheniformis* and *Bacillus subtilis* on Gut Barrier Function, Proinflammatory Response, ROS Production and Pathogen Inhibition Properties in IPEC-J2—*Escherichia coli*/*Salmonella* Typhimurium Co-Culture

**DOI:** 10.3390/microorganisms10050936

**Published:** 2022-04-29

**Authors:** Nikolett Palkovicsné Pézsa, Dóra Kovács, Bence Rácz, Orsolya Farkas

**Affiliations:** 1 Department of Pharmacology and Toxicology, University of Veterinary Medicine Budapest, 1078 Budapest, Hungary; kovacs.dora@univet.hu (D.K.); farkas.orsolya@univet.hu (O.F.); 2 Department of Anatomy and Histology, University of Veterinary Medicine Budapest, 1078 Budapest, Hungary; racz.bence@univet.hu

**Keywords:** *Bacillus licheniformis*, *Bacillus subtilis*, *Escherichia coli*, *Salmonella* Typhimurium, IPEC-J2, barrier function, ROS, proinflammatory cytokines, adhesion, antibiotic alternative

## Abstract

The emergence of antimicrobial resistance raises serious concerns worldwide. Probiotics offer a promising alternative to enhance growth promotion in farm animals; however, their mode of action still needs to be elucidated. The IPEC-J2 cell line (porcine intestinal epithelial cells) is an appropriate tool to study the effect of probiotics on intestinal epithelial cells. In our experiments, IPEC-J2 cells were challenged by two gastrointestinal (GI) infection causing agents, *Escherichia coli* (*E. coli*) or *Salmonella enterica* ser. Typhimurium (*S.* Typhimurium). We focused on determining the effect of pre-, co-, and post-treatment with two probiotic candidates, *Bacillus licheniformis* or *Bacillus subtilis*, on the barrier function, proinflammatory cytokine (IL-6 and IL-8) response, and intracellular reactive oxygen species (ROS) production of IPEC-J2 cells, in addition to the adhesion inhibition effect. *Bacillus licheniformis* (*B. licheniformis)* and *Bacillus subtilis* (*B. subtilis)* proved to be anti-inflammatory and had an antioxidant effect under certain treatment combinations, and further effectively inhibited the adhesion of pathogenic bacteria. Interestingly, they had little effect on paracellular permeability. Based on our results, *Bacillus licheniformis* and *Bacillus subtilis* are both promising candidates to contribute to the beneficial effects of probiotic multispecies mixtures.

## 1. Introduction

The demands for food of the growing human population have urged for the intensification of food animal production. In food-producing animals, growth promotion was reached for decades with the prophylactic use of antibiotics [1]. However, the misuse of antimicrobials, leading to the emergence of antimicrobial resistance, residues in human food, and environmental pollution, has raised serious concerns worldwide [2,3]. The One Health concept offers a comprehensive approach to tackle this problem [4]. Measures have also been taken to restrict the use of antibiotics in veterinary medicine [5]. However, to ensure growth performance, it has become an important research issue to all food animal-producing sectors—including the swine and poultry industry—to find alternatives that are capable of maintaining the health of the gastrointestinal tract [6]. Among enzymes, phytochemicals, organic acids, antimicrobial peptides, anti-bacterial virulence drugs, minerals, bacteriophages, and probiotics (defined as “*live microorganisms which when administered in adequate amounts confer a health benefit on the host*”) are attractive non-invasive candidates [7,8]. Several studies emphasize that probiotics may be the most promising choice among alternative feed additives, and their beneficial effects in several animal species have indeed been demonstrated recently [1,2,3,9,10,11,12,13]. Probiotic action is complex; among the beneficial effects exerted by probiotics are stimulation of heat shock proteins, induction of cytokine production, antioxidant properties, enhancement of barrier function, and inhibition of pathogen adhesion and proliferation [14,15,16,17,18,19,20,21,22]. The exact mechanism of probiotic action has been widely studied in many species such as humans [23], companion animals [24], poultry [6], and swine [14,25]. Once the underlying mechanism of probiotic action in pigs is fully understood, conclusions could be extended—with certain limitations—to human application owing to the similarity between the human and swine gut [26].

Most probiotic bacteria belong to the genera *Bifidobacterium*, *Lactobacillus*, and *Enterococcus* and originate from the intestine [14,27]. *Bacillus* strains are not part of the commensal flora, but are also attractive probiotic candidates thanks to their spore forming properties, which them enable to resist during the transit through the gastrointestinal tract [25,27]. Further advantages of spores are good reproducibility, high viability, and stability during storage and feed preparation processes. *Bacillus* spp. form biofilms; survive and germinate in the gut; and have several modes of action, which can affect the health of the gastrointestinal tract, stimulate the immune system, contribute to feed efficiency, have a direct effect on pathogens, and support the colonization of beneficial bacteria—these are the most important ones [2,28]. Among various *Bacillus* spp. strains, *B. subtilis*, *B. licheniformis*, and *B. cereus* are used for animal feed [28]. *B. licheniformis* and *B. subtilis* also have industrial relevance, as they produce compounds such as enzymes, amino acids, vitamins, and other substances essential for the food industry and biofuel production [29]. However, among *Bacillus* species, pathogenic members can also be found, which raises general concern about their use as probiotics [30]. The production of enterotoxins and the possible transfer of antibiotic resistance genes might further contribute to their limited use. [2]

In vivo studies showed that *Bacillus* strains were successful in reducing diarrhea in post-weaning pigs [2,31,32], and immune responses were enhanced upon pathogen (enterotoxigenic *E. coli* K88) challenge in weaned pigs [3]. Supplementation of *Bacillus* strains also had beneficial effects on productive parameters such as growth performance and feed efficiency [2,3,33,34,35].

The use of cell lines is recommended by the 3R (reduction, replacement, refinement) concept, according to which experiments conducted on animals should be reduced, replaced, and refined [36]. The IPEC-J2 cell line is of piglet jejunum origin; is non tumorigenic in nature; and is well characterized, which makes it an appropriate tool to study host–microbe interactions, immune responses, and the effect of many substances on intestinal epithelial cells [19,26,37,38,39]. Furthermore, it can also mimic the gastro intestinal tract (GIT) of humans thanks to the similar structure of human and pig intestines [26]. The IPEC-J2 cell line is widely used to study the effects of probiotic bacteria [40,41,42].

The objective of this study was to examine the probiotic properties of *Bacillus licheniformis* and *Bacillus subtilis,* including alteration of paracellular permeability, antioxidant, and anti-inflammatory effects in response to pathogen (*S.* Typhimurium or *E. coli*) challenge. Adhesion inhibition of pathogens to IPEC-J2 cells was also investigated.

## 2. Materials and Methods

### 2.1. Bacteria for Cell Culture Challenge

*S.* Typhimurium and *E. coli* originated from GI infections in pigs and were isolates from clinical samples in Hungary (obtained from the Department of Microbiology and Infectious Diseases, University of Veterinary Medicine Budapest). Identification was verified by the Department of Microbiology and Infectious Diseases. *E. coli* expresses F4 fimbriae, and produces both heat-stable (STa and STb) and heat-labile (LT) enterotoxins. *Bacillus licheniformis* and *Bacillus subtilis* were acquired from the Hungarian Diary Experimental Institute Ltd. and were also swine intestine isolates. All four bacterial strains were preserved on Microbank beads at −80 °C.

Cell suspensions were prepared by suspending microbeads in plain DMEM/F12 (without supplementation). Incubation was performed for 18–24 h at 37 °C in the presence of 5% CO_2_/95% air atmosphere in order to mimic culture conditions of IPEC-J2 cells. In previous experiments, *B. licheniformis*, *B. subtilis*, *E. coli*, and *S.* Typhimurium were shown to grow to 10^8^ CFU/mL under these circumstances. In the pre-, co-, and post-treatment solutions, the applied concentration of *Bacillus licheniformis* and *Bacillus subtilis* was 10^8^ CFU/mL. *E. coli* and *S.* Typhimurium suspensions were diluted from the stock solutions to 10^6^ CFU/mL using plain DMEM/F12 medium (free of antibiotics) as a dilution reagent.

### 2.2. Cell Line and Culture Conditions

The IPEC-J2 epithelial cell line was a kind gift from Dr. Jody Gookin’s Department of Clinical Sciences, College of Veterinary Medicine, North Carolina State University, Raleigh, NC, USA. IPEC-J2 cells were grown and maintained in a complete medium consisting of 10 mL of Dulbecco’s modified Eagle’s medium and Ham’s F-12 nutrient mixture (DMEM/F12) in a 1:1 ratio, supplemented with 5% foetal bovine serum (FBS), 5 μg/mL insulin, 5 μg/mL transferrin, 5 ng/mL selenium, 5 ng/mL epidermal growth factor (EGF), and 1% penicillin-streptomycin (Biocenter Ltd., Szeged, Hungary). Cells were cultured at 37 °C in 5% CO_2_ atmosphere. [37]. Cells with a passage number 49–52 were used for our experiments. For IL-6, IL-8, and intracellular ROS determination, cells were grown on 6-well culture plates (Costar Corning INc., Corning, NY, USA); for adhesion inhibition assays, cells were seeded onto 24-well cell culture plates (Costar Corning INc., Corning, NY, USA); and for the measurement of paracellular permeability, cells were cultured on 12-well polyester membrane cell culture inserts (Costar Corning Inc., Corning, NY, USA) until confluency was reached.

In order to remove the remaining antibiotics before starting the treatment of IPEC-J2 cells with the different treatment solutions (described in Section 2.1), IPEC-J2 cells were washed twice with PBS, and then DMEM/F12 without antibiotics was added to each well and cells were incubated for 30 min at 37 °C.

### 2.3. Experimental Setup

**Figure 1:**
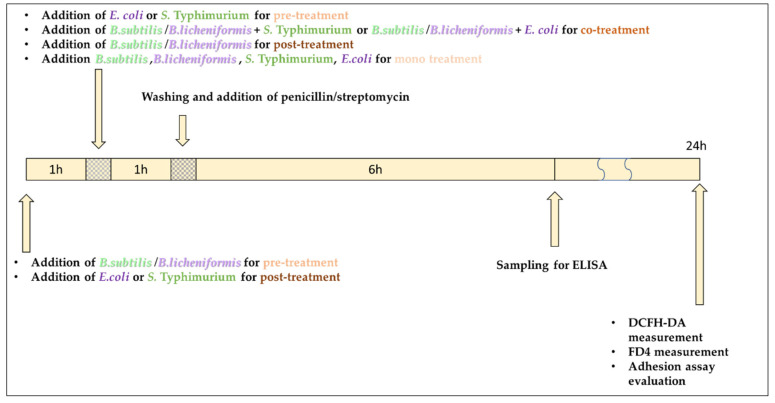
Timeline for experimental setup. DCFH-DA: 2′,7′-dichloro-dihydro-fluorescein diacetate dye; FD4: fluorescein isothiocyanate–dextran tracer dye.

In our experiments, IPEC-J2 cells were incubated for 1 h with the pathogen strain *E. coli* or *S.* Typhimurium, respectively. Control cells received plain DMEM/F12 medium. As a positive control, IPEC-J2 cells were mono-incubated with only *E. coli* (10^6^ CFU/mL) or *S.* Typhimurium (10^6^ CFU/mL), respectively. The influence of *E. coli* and *S.* Typhimurium suspensions applied in different concentrations and for different incubation periods was tested previously by our research group [38]. For pre-treatment assays, cells were pre-incubated with *B. subtilis* or *B. licheniformis* for 1 h before the addition of the pathogen strain. For co-treatment experiments, the pathogen strain (*E. coli* or *S.* Typhimurium) and *B. subtilis* or *B. licheniformis* was added at the same time to IPEC-J2 cells. In our post-treatment assay, IPEC-J2 cells were incubated with *B. subtilis* or *B. licheniformis* for 1 h after the treatment with the pathogen strains (*E. coli* or *S*. Typhimurium). Bacterial infections were performed with *E. coli* or *S.* Typhimurium at a concentration of 10^6^ CFU/mL. *B. subtilis* or *B. licheniformis* suspensions were applied at a concentration of 10^8^ CFU/ mL. IPEC-J2 cells were also mono-incubated with *B. subtilis* or *B. licheniformis* at 10^8^ CFU/mL. If further incubation was needed after the treatments, cells were washed with PBS and DMEM/F12 supplemented with antibiotics. Then, 1% penicillin-streptomycin was added to prevent the growth of bacteria. Figure 1 shows the timeline of our experimental setup.

### 2.4. Paracellular Permeability Measurements/Assay

The effect of *B. subtilis/B. licheniformis* and *E. coli* or *S.* Typhimurium on the paracellular permeability of IPEC-J2 cells was evaluated with fluorescein isothiocyanate–dextran (FD4) tracer dye (Sigma-Aldrich, Darmstadt, Germany). Prior to treatments, transepithelial electrical resistance (TEER) values of IPEC-J2 cells were measured to check the development of a differentiated, confluent monolayer. Mono-, pre-, co-, and post-treatments were performed as described in the experimental setup section. After treatment, the cells were washed with PBS, and FD4 (dissolved in fenol free DMEM/F12 medium) at a final concentration of 0.25 mg/mL was added to the apical layer cells. To the basolateral chamber, phenol-free DMEM/F12 medium was added. Cells were incubated at 37 °C (5% CO_2_). Samples of 100 μL were taken from the basolateral chamber after 24 h. The fluorescent signal was measured with a Spectramax iD3 instrument (Molecular Devices, San Jose, CA, USA) using 485 nm excitation and 535 nm emission wavelength.

### 2.5. IL-6 and IL-8 Determination with ELISA

For the enzyme-linked immunosorbent assay (ELISA), experiments cells were seeded onto six-well culture plates and pre-, co-, and post-treatments were performed as described in the experimental setup section. After the removal of treatment solutions, IPEC-J2 cells were incubated with cell culture medium and cell supernatants were collected after 6 h. IL-6 and IL-8 secretion was determined by porcine-specific ELISA Kits (Sigma-Aldrich, Darmstadt, Germany) according to the manufacturer’s instructions.

### 2.6. Determination of ROS Production of IPEC-J2 Cells

To evaluate the effect of *B. subtilis and B. licheniformis* on the intracellular ROS production of IPEC-J2 cells, the DCFH-DA method was used. The 2′,7′-dichloro-dihydro-fluorescein diacetate (DCFH-DA) dye (Sigma-Aldrich, Budapest, Hungary) is oxidized to the highly fluorescent form dichloro- fluorescein (DCF) by the intracellular ROS [43]. In IPEC-J2 cells, inflammation was evoked by *E. coli* (10^6^ CFU/mL) or *S.* Typhimurium (10^6^ CFU/mL), respectively. *B. subtilis or B. licheniformis* (10^8^ CFU/mL) was added as pre-, co-, or post-treatment. Moreover, the effect of *B. subtilis* and *B. licheniformis* alone (applied in 10^8^ CFU/mL) on the amount of intracellular reactive oxygen species was tested. Cells treated with plain medium were used as a negative control and cells treated with either *E. coli* or *S.* Typhimurium served as positive controls. After the treatment, the treatment solutions were discarded and plain medium containing 1% penicillin-streptomycin was added.

For the detection, the cells were washed with PBS after 24 h, and DCFH-DA reagent (40 mM) was added to the cells. After one hour, the reagent was removed, cells were washed twice with phenol-free plain DMEM/F12 (2 mL), and the cells were scraped and lysed. The lysed cells were then pipetted into an Eppendorf tube and centrifuged for 10 min at 4 °C at 4500 rpm. Then, 100 μL of supernatant from each sample was added to a 96-well plate. A Spectramax iD3 instrument was used to measure the fluorescence at an excitation wavelength of 480 nm and an emission wavelength of 530 nm.

### 2.7. Adhesion Inhibition Assay

In order to evaluate the inhibitory effect of *B. subtilis* or *B. licheniformis* on *E. coli* or *S.* Typhimurium adhesion to IPEC-J2 cells, *B. subtilis* or *B. licheniformis* was added at 10^8^ CFU/mL as pre-, co-, or post-treatment. As control, cells treated with only *E. coli* or *S.* Typhimurium were used. IPEC-J2 cells were incubated for 1 h and then washed to remove unbound bacteria. The lysis of cells was performed with 500 µL 0.1% Triton X-100 (Sigma-Aldrich, Darmstadt, Germany). Viable *E. coli* and *S.* Typhimurium counts were determined by serial dilution and plating on ChromoBio Coliform (for *E. coli*) or ChromoBio *Salmonella* Plus Base (for *S.* Typhimurium) agar. ChromoBio Coliform and ChromoBio *Salmonella* Plus Base selective agars were purchased from Biolab Zrt. (Budapest, Hungary). Adhesion was calculated as a control percentage. Adhering *E. coli* and *S.* Typhimurium were normalized to the control.

### 2.8. Statistical Analysis

Statistical analysis of the data obtained in the cell culture experiments was performed with R 4.0.4 software (R Foundation for Statistical Computing, Vienna, Austria) package. Differences among the mean values of different experimental groups were evaluated with one-way ANOVA and Tukey post-hoc test. The results were interpreted as significant if the *p*-value was lower than 0.05.

## 3. Results

### 3.1. The Effect of Bacillus subtilis and Bacillus licheniformis on Paracellular Permeability of IPEC-J2 Cells Challenged by E. coli and S. Typhimurium

**Figure 2:**
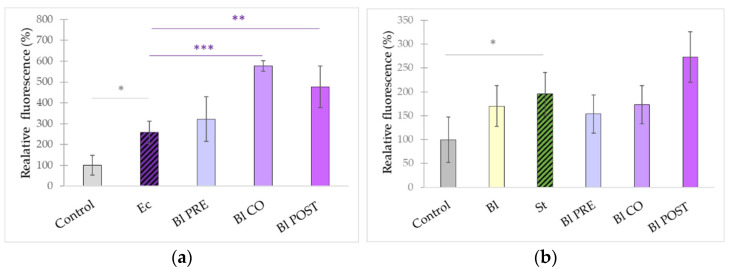
Effect of *B. licheniformis* on the paracellular permeability of IPEC-J2 cells treated with *E. coli* (**a**) and *S.* Typhimurium (**b**), respectively. *B. licheniformis* was added 1 h before (pre-treatment), at the same time as (co-treatment), and 1 h after (post-treatment) the addition of *S.* Typhimurium or *E. coli*, respectively. Detection of the FD4 dye was performed 24 h after the treatment of *S.* Typhimurium or *E. coli,* respectively. **Control**: plain cell culture medium treatment; **Ec**: *E. coli* 10^6^ CFU/mL; St: *S.* Typhimurium 10^6^ CFU/mL; **Bl**: treatment with *B. licheniformis* 10^8^ CFU/mL; **Bl PRE**: pre-treatment with *B. licheniformis* 10^8^ CFU/mL + *E. coli* 10^6^ or *S.* Typhimurium CFU/mL; **Bl CO**: co-treatment with *B. licheniformis* 10^8^ CFU/mL + *E. coli* or *S.* Typhimurium 10^6^ CFU/mL; **Bl POST**: post-treatment with *B. licheniformis* 10^8^ CFU/mL + *E. coli* or *S.* Typhimurium 10^6^ CFU/mL. Data are shown as means with standard deviations and expressed as relative fluorescence, considering the mean value of control as 100%. n = 6/group. Significant difference: * *p* ≤ 0.05; in grey: compared with the untreated control. ** *p* ≤ 0.01; *** *p* ≤ 0.0001, in purple: compared with treatment with *E. coli*.

After 24 h of pathogen exposure, the epithelial cell layer was partially disrupted. Fluorescence intensity measured in the basolateral compartment significantly increased (compared with untreated control samples) when IPEC-J2 cells were treated with *S.* Typhimurium (Figure 2a) or *E. coli* (Figure 2b). The treatment with *B. licheniformis* alone did not result in the alteration of fluorescence intensity (Figure 2b). None of the treatments could significantly decrease the presence of FD4 tracer in the basolateral chamber. However, in the cases of co- and post-treatment with *B. licheniformis*, fluorescence intensity was further significantly increased compared with the fluorescence when IPEC-J2 cells were challenged by *E. coli* (Figure 2a).

**Figure 3:**
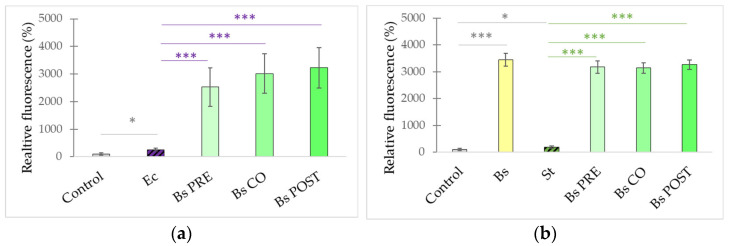
Effect of *B. subtilis* on the paracellular permeability of IPEC-J2 cells treated with *E. coli* (**a**) and *S.* Typhimurium (**b**), respectively. *B. subtilis* was added 1 h before (pre-treatment), at the same time as (co-treatment), and 1 h after (post-treatment) the addition of *S.* Typhimurium or *E. coli*, respectively. Detection of the FD4 dye was performed 24 h after the treatment of *S.* Typhimurium or *E. coli,* respectively. **Control**: plain cell culture medium treatment; **Ec**: *E. coli* 10^6^ CFU/mL; **St**: *S.* Typhimurium 10^6^ CFU/mL; **Bs**: treatment with *B. subtilis* 10^8^ CFU/mL; **Bs PRE**: pre-treatment with *B. subtilis* 10^8^ CFU/mL + *E. coli* 10^6^ or *S.* Typhimurium CFU/mL; **Bs CO**: co-treatment with *B. subtilis* 10^8^ CFU/mL + *E. coli* or *S.* Typhimurium 10^6^ CFU/mL; **Bs POST**: post-treatment with *B. subtilis* 10^8^ CFU/mL + *E. coli* or *S.* Typhimurium 10^6^ CFU/mL. Data are shown as means with standard deviations and expressed as relative fluorescence, considering the mean value of control as 100%. n = 6/group. Significant difference: * *p* ≤ 0.05,*** *p* ≤ 0.0001, in grey: compared with the untreated control. *** *p* ≤ 0.0001, in green: compared with treatment with *S.* Typhimurium, *** *p* ≤ 0.0001, in purple: compared with treatment with *E. coli*.

Treatment with *B. subtilis* alone caused an increase in paracellular permeability compared with the control (Figure 3b). Pre-, co-, and post-treatments further increased the fluorescence signal measured in the basolateral compartment compared with the fluorescence intensity increase induced by pathogens (Figure 3a,b).

### 3.2. Effect of Bacillus subtilis and Bacillus Licheniformis on IL-6 and IL-8 Production of IPEC-J2 Cells Provoked by E. coli or S. Typhimurium

**Figure 4:**
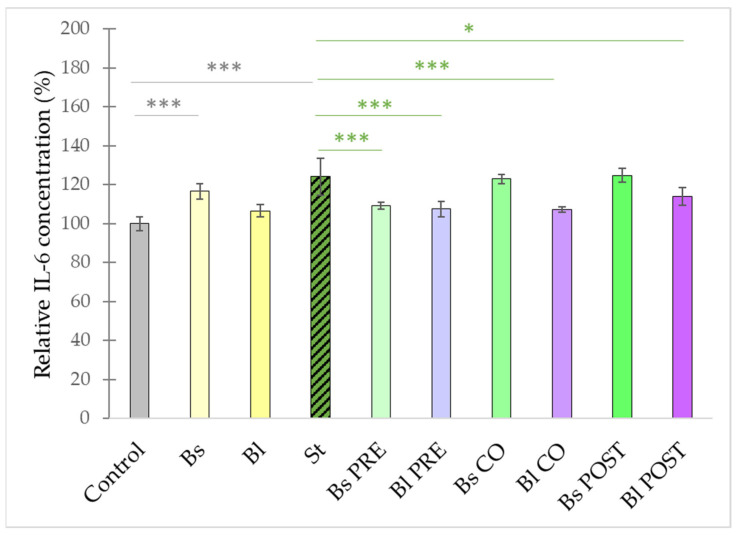
Induction of IL-6 secretion of IPEC-J2 cells after stimulation with *S.* Typhimurium, *B. licheniformis,* and *B. subtilis*. *B. licheniformis* and *B. subtilis* were added 1 h before (pre-treatment), at the same time as (co-treatment), or 1 h after (post-treatment) the addition of *S.* Typhimurium. *B. licheniformis* and *B. subtilis* were added in 10^8^ CFU/mL and *S.* Typhimurium was added in 10^6^ CFU/mL concentration. **Control**: plain cell culture medium treatment; **St**: *S.* Typhimurium 10^6^ CFU/mL; **Bs**: *B. subtilis* 10^8^ CFU/mL; **Bl**: *B. licheniformis* 10^8^ CFU/mL; **Bs PRE**: pre-treatment with *B. subtilis* 10^8^ CFU/mL + *S.* Typhimurium 10^6^ CFU/mL; **Bl PRE**: pre-treatment with *B. licheniformis* 10^8^ CFU/mL + *S.* Typhimurium 10^6^ CFU/mL; **Bs CO**: co-treatment with *B. subtilis* 10^8^ CFU/mL + *S.* Typhimurium 10^6^ CFU/mL; **Bl CO**: co-treatment with *B. licheniformis* 10^8^ CFU/mL + *S.* Typhimurium 10^6^ CFU/mL; **Bs POST**: post-treatment with *B. subtilis* 10^8^ CFU/mL + *S.* Typhimurium 10^6^ CFU/mL; **Bl POST**: post-treatment with *B. licheniformis* 10^8^ CFU/mL + *S.* Typhimurium 10^6^ CFU/mL. Data are shown as means with standard deviations and expressed as relative IL-6 concentration, considering the mean value of control as 100%. n = 6/group. Significant difference: *** *p* ≤ 0.0001, in grey: compared with the untreated control. * *p* ≤ 0.05; *** *p* ≤ 0.0001, in green: compared with treatment with *S.* Typhimurium.

Infection of intestinal epithelial cells with *S.* Typhimurium significantly induced the secretion of IL-6 compared with controls (i.e., non-infected cells) (Figure 4). The treatment with *B. subtilis* alone also resulted in significant IL-6 secretion compared with the control. In comparison, treatment with only *B. licheniformis* did not result in a significant change in IL-6 secretion compared with the control. The pre-treatment with both *B. subtilis* 10^8^ CFU/mL and *B. licheniformis* 10^8^ CFU/mL caused a significant decrease in IL-6 production as compared with the IL-6 secretion induced by *S.* Typhimurium. The co- and post-treatments with *B. licheniformis* 10^8^ CFU/mL also reduced the IL-6 secretion; however, the co- and post-treatments with *B. subtilis* 10^8^ CFU/mL failed to significantly decrease IL-6 secretion compared with the IL-6 production induced by *S.* Typhimurium.

**Figure 5:**
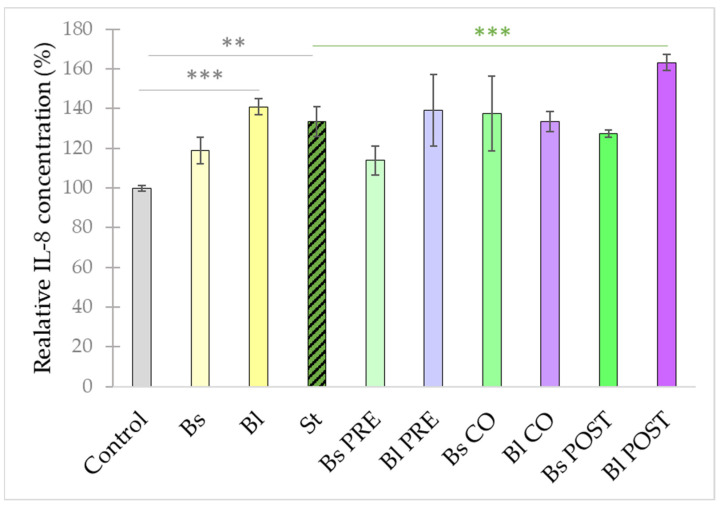
Induction of IL-8 secretion of IPEC-J2 cells after stimulation with *S.* Typhimurium, *B. licheniformis,* and *B. subtilis*. *B. licheniformis* and *B. subtilis* were added 1 h before (pre-treatment), at the same time as (co-treatment), or 1 h after (post-treatment) the addition of *S.* Typhimurium. *B. licheniformis* and *B. subtilis* were added in 10^8^ CFU/mL and *S.* Typhimurium was added in 10^6^ CFU/mL concentration. **Control**: plain cell culture medium treatment; **St**: *S.* Typhimurium 10^6^ CFU/mL; **Bs**: *B. subtilis* 10^8^ CFU/mL; **Bl**: *B. licheniformis* 10^8^ CFU/mL; **Bs PRE**: pre-treatment with *B. subtilis* 10^8^ CFU/mL + *S.* Typhimurium 10^6^ CFU/mL; **Bl PRE**: pre-treatment with *B. licheniformis* 10^8^ CFU/mL + *S.* Typhimurium 10^6^ CFU/mL; **Bs CO**: co-treatment with *B. subtilis* 10^8^ CFU/mL + *S.* Typhimurium 10^6^ CFU/mL; **Bl CO**: co-treatment with *B. licheniformis* 10^8^ CFU/mL + *S.* Typhimurium 10^6^ CFU/mL; **Bs POST**: post-treatment with *B. subtilis* 10^8^ CFU/mL + *S.* Typhimurium 10^6^ CFU/mL; **Bl POST**: post-treatment with *B. licheniformis* 10^8^ CFU/mL + *S.* Typhimurium 10^6^ CFU/mL. Data are shown as means with standard deviations and expressed as relative IL-8 concentration, considering the mean value of control as 100%. n = 6/group. Significant difference: ** *p* ≤ 0.01, *** *p* ≤ 0.0001, in grey: compared with the untreated control *** *p* ≤ 0.0001, in green: compared with treatment with *S.* Typhimurium.

Infection of IPEC-J2 cells with *S.* Typhimurium also triggered the secretion of IL-8 (Figure 5). Treatment with *B. licheniformis* alone also resulted in a significant rise in IL-8 secretion compared with the control. However, the treatment with *B. subtilis* alone did not result in a significant change in IL-8 secretion compared with the control. With the exception of post-treatment with *B. licheniformis*, all other treatment combinations did not alter the IL-8 secretion induced by *S.* Typhimurium. Post-treatment with *B. licheniformis* further increased the IL-8 secretions compared with the amount of IL-8 secretion when IPEC-J2 cells were challenged by *S.* Typhimurium.

**Figure 6:**
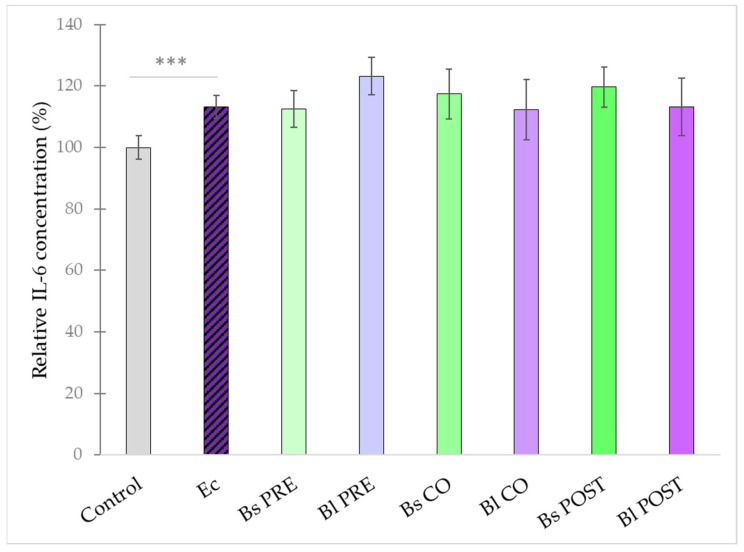
Induction of IL-6 secretion of IPEC-J2 cells after stimulation with *E. coli*, *B. licheniformis,* and *B. subtilis*. *B. licheniformis* and *B. subtilis* were added 1 h before (pre-treatment), at the same time as (co-treatment), or 1 h after (post-treatment) the addition of *E. coli*. *B. licheniformis* and *B. subtilis* were added in 10^8^ CFU/mL and *E. coli* was added in 10^6^ CFU/mL concentration. **Control**: plain cell culture medium treatment; **Ec**: *E. coli* 10^6^ CFU/mL; **Bs PRE**: pre-treatment with *B. subtilis* 10^8^ CFU/mL + *E. coli* 10^6^ CFU/mL; **Bl PRE**: pre-treatment with *B. licheniformis* 10^8^ CFU/mL + *E. coli* 10^6^ CFU/mL; **Bs CO**: co-treatment with *B. subtilis* 10^8^ CFU/mL + *E. coli* 10^6^ CFU/mL; **Bl CO**: co-treatment with *B. licheniformis* 10^8^ CFU/mL + *E. coli* 10^6^ CFU/mL; **Bs POST**: post-treatment with *B. subtilis* 10^8^ CFU/mL + *E. coli* 10^6^ CFU/mL; **Bl POST**: post-treatment with *B. licheniformis* 10^8^ CFU/mL + *E. coli* 10^6^ CFU/mL. Data are shown as means with standard deviations and expressed as relative IL-6 concentration, considering the mean value of control as 100%. n = 6/group. Significant difference: *** *p* ≤ 0.0001 in grey: compared with the untreated control.

IL-6 secretion was induced significantly by *E. coli* in comparison with control cells. None of the pre-, co-, and post-treatments with *B. licheniformis* and *B. subtilis* had any significant effect on the IL-6 elevation induced by *E. coli* (Figure 6).

**Figure 7:**
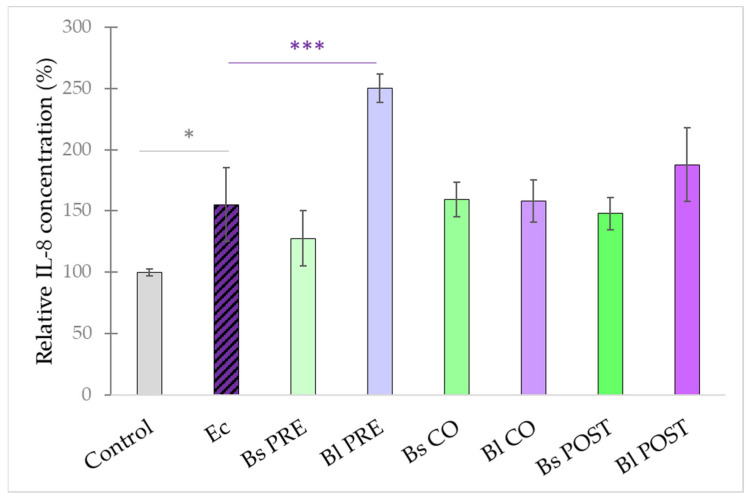
Induction of IL-8 secretion of IPEC-J2 cells after stimulation with *E. coli*, *B. licheniformis,* and *B. subtilis*. *B. licheniformis* and *B. subtilis* were added 1 h before (pre-treatment), at the same time as (co-treatment), or 1 h after (post-treatment) the addition of *E. coli*. *B. licheniformis* and *B. subtilis* were added in 10^8^ CFU/mL and *E. coli* was added in 10^6^ CFU/mL concentration. **Control**: plain cell culture medium treatment; **Ec**: *E. coli* 10^6^ CFU/mL; **Bs PRE**: pre-treatment with *B. subtilis* 10^8^ CFU/mL + *E. coli* 10^6^ CFU/mL; **Bl PRE**: pre-treatment with *B. licheniformis* 10^8^ CFU/mL + *E. coli* 10^6^ CFU/mL; **Bs CO**: co-treatment with *B. subtilis* 10^8^ CFU/mL + *E. coli* 10^6^ CFU/mL; **Bl CO**: co-treatment with *B. licheniformis* 10^8^ CFU/mL + *E. coli* 10^6^ CFU/mL; **Bs POST**: post-treatment with *B. subtilis* 10^8^ CFU/mL + *E. coli* 10^6^ CFU/mL; **Bl POST**: post-treatment with *B. licheniformis* 10^8^ CFU/mL + *E. coli* 10^6^ CFU/mL. Data are shown as means with standard deviations and expressed as relative IL-8 concentration, considering the mean value of control as 100%. n = 6/group. Significant difference: * *p* ≤ 0.05, in grey: compared with the untreated control. *** *p* ≤ 0.0001, in purple: compared with treatment with *E. coli*.

IL-8 secretion was induced significantly by *E. coli* compared with control cells (Figure 7), and pre-treatment with *B. licheniformis* 10^8^ CFU/mL further increased the secretion of IL-8. Pre-treatment with *B. subtilis* and co- and post-treatments with both probiotic bacteria failed to cause any significant effect on IL-8 secretion.

### 3.3. Effect of Bacillus subtilis and Bacillus licheniformis on the Intracellular Redox State of IPEC-J2 Cells Challenged by Salmonella Typhimurium and Escherichia coli

**Figure 8:**
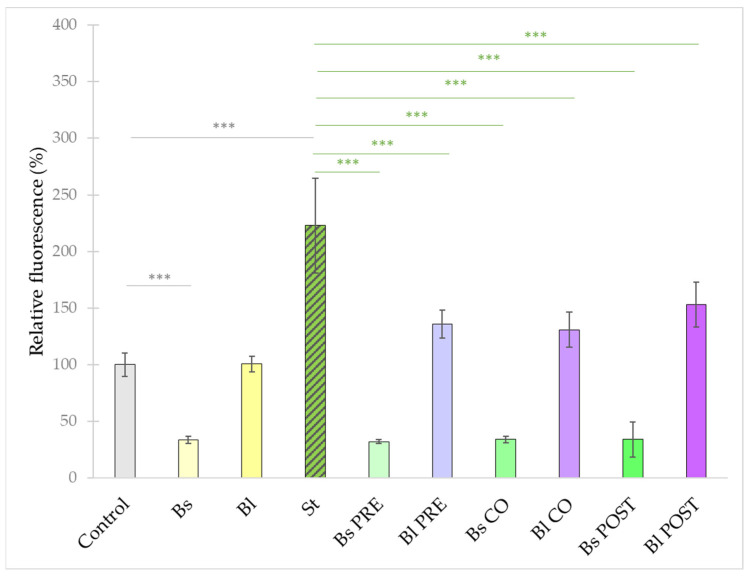
Amount of intracellular ROS after treatment with *S.* Typhimurium, *B. licheniformis,* and *B. subtilis* and their combinations. *B. licheniformis* and *B. subtilis* were added 1 h before (pre-treatment), at the same time as (co-treatment), or after (post-treatment) the addition of *S.* Typhimurium. **Control**: plain cell culture medium treatment; **St**: *S.* Typhimurium 10^6^ CFU/mL; **Bs**: *B. subtilis* 10^8^ CFU/mL; **Bl**: *B. licheniformis* 10^8^ CFU/mL; **Bs PRE**: pre-treatment with *B. subtilis* 10^8^ CFU/mL + *S.* Typhimurium 10^6^ CFU/mL; **Bl PRE**: pre-treatment with *B. licheniformis* 10^8^ CFU/mL + *S.* Typhimurium 10^6^ CFU/mL; **Bs CO**: co-treatment with *B. subtilis* 10^8^ CFU/mL + *S.* Typhimurium 10^6^ CFU/mL; **Bl CO**: co-treatment with *B. licheniformis* 10^8^ CFU/mL + *S.* Typhimurium 10^6^ CFU/mL; **Bs POST**: post-treatment with *B. subtilis* 10^8^ CFU/mL + *S.* Typhimurium 10^6^ CFU/mL; **Bl POST**: post-treatment with *B. licheniformis* 10^8^ CFU/mL + *S.* Typhimurium 10^6^ CFU/mL. Data are shown as means with standard deviations and expressed as relative fluorescence, considering the mean value of control as 100%. n = 6/group. Significant difference: *** *p* ≤ 0.0001, in grey: compared with the untreated control. *** *p* ≤ 0.0001, in green: compared with treatment with *S.* Typhimurium.

In order to characterize the intracellular redox state of the IPEC-J2 cells, the DCFH-DA method was used. Treatment with *S.* Typhimurium caused an increase in the fluorescence compared with the control (Figure 8). Treatment with *B. subtilis* alone significantly decreased the fluorescence compared with the control; however, when IPEC-J2 cells were treated with only *B. licheniformis*, no significant effect compared with the control could be observed. Pre-, co-, and post-treatment with both probiotic bacteria resulted in a decreased amount of ROS compared with ROS production induced by *S.* Typhimurium.

**Figure 9:**
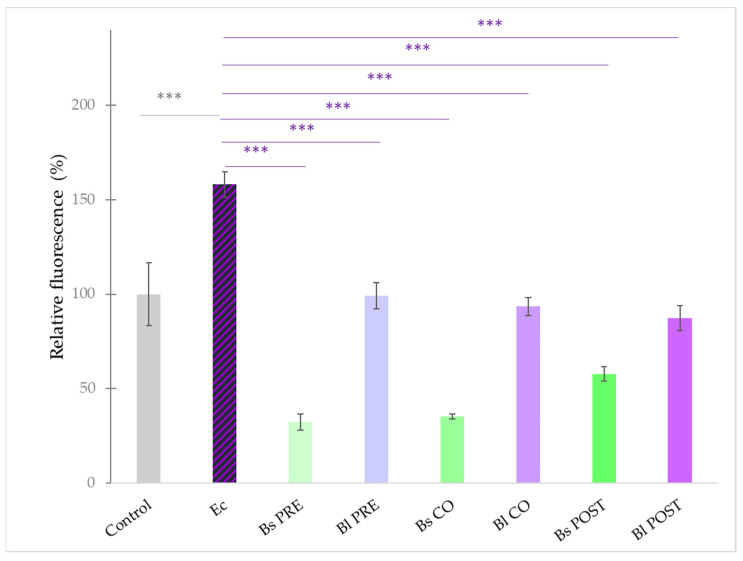
Amount of intracellular ROS after treatment with *E. coli*, *B. licheniformis,* and *B. subtilis* and their combinations. *B. licheniformis* and *B. subtilis* were added 1 h before (pre-treatment), at the same time as (co-treatment), or after (post-treatment) the addition of *E. coli*. **Control**: plain cell culture medium treatment; **Ec**: *E. coli* 10^6^ CFU/mL; **Bs PRE**: pre-treatment with *B. subtilis* 10^8^ CFU/mL + *E. coli* 10^6^ CFU/mL; **Bl PRE**: pre-treatment with *B. licheniformis* 10^8^ CFU/mL + *E. coli* 10^6^ CFU/mL; **Bs CO**: co-treatment with *B. subtilis* 10^8^ CFU/mL + *E. coli* 10^6^ CFU/mL; **Bl CO**: co-treatment with *B. licheniformis* 10^8^ CFU/mL + *E. coli* 10^6^ CFU/mL; **Bs POST**: post-treatment with *B. subtilis* 10^8^ CFU/mL + *E. coli* 10^6^ CFU/mL; **Bl POST**: post-treatment with *B. licheniformis* 10^8^ CFU/mL + *E. coli* 10^6^ CFU/mL. Data are shown as means with standard deviations and expressed as relative fluorescence, considering the mean value of control as 100%. n = 6/group. Significant difference: *** *p* ≤ 0.0001, in grey: compared with the untreated control. *** *p* ≤ 0.0001, in purple: compared with treatment with *E. coli*.

Treatment with *E. coli* caused an increase in the fluorescence compared with the control (Figure 9). Pre-, co-, and post-treatment with both probiotic bacteria significantly reduced the amount of reactive oxygen species in the cells compared with samples only treated with *E. coli*.

### 3.4. Effect of Bacillus subtilis and Bacillus licheniformis on the Adhesion of S. Typhimurium and E. coli to IPEC-J2 Cells

**Figure 10:**
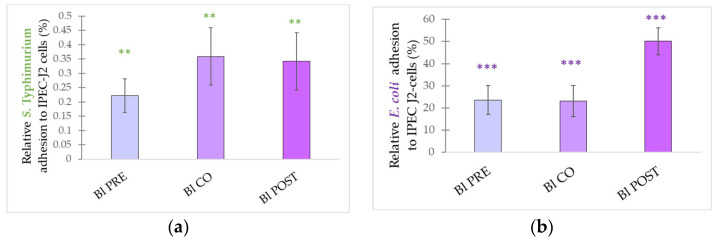
Inhibitory effect of *B. licheniformis* on *S.* Typhimurium (**a**) and *E. coli* (**b**) adhesion to IPEC-J2 cells. *S.* Typhimurium and *E. coli* adhesion inhibitions were determined upon incubation with *B. licheniformis* added 1 h before (pre-treatment), at the same time as (co-treatment), and 1 h after (post-treatment) the addition of *S.* Typhimurium and *E. coli*, respectively. *B. licheniformis* was added in 10^8^ CFU/mL. **Bl PRE**: pre-treatment with *B. licheniformis* 10^8^ CFU/mL + *E. coli* or *S.* Typhimurium 10^6^ CFU/mL; **Bl CO**: co-treatment with *B. licheniformis* 10^8^ CFU/mL + *E. coli* or *S.* Typhimurium 10^6^ CFU/mL; **Bl POST**: post-treatment with *B. licheniformis* 10^8^ CFU/mL + *E. coli* or *S.* Typhimurium 10^6^ CFU/mL. Data are shown as means with standard deviations and expressed as relative adhesion, considering the mean value of only *S.* Typhimurium or *E. coli* treated cells as 100%. n = 6/group. Significant difference: ** *p* ≤ 0.01, in green: compared with treatment with *S.* Typhimurium. *** *p* ≤ 0.0001, in purple: compared with treatment with *E. coli*.

*B. licheniformis* was able to inhibit the adhesion of both *E. coli* and *S.* Typhimurium in all treatment combinations (Figure 10a,b). When IPEC-J2 cells were challenged by *S.* Typhimurium, pre-treatment with *B. licheniformis* had the highest inhibitory effect, followed by post-treatment, while co-treatment showed the lowest inhibitory effect. *S.* Typhimurium adhesion was 0.22% in the case of pre-treatment, 0.34% in the post-treatment assay, and 0.35% for the co-treatment. When IPEC-J2 cells were exposed to *E. coli*, pre-treatment and co-treatment had almost the same effect, while post-treatment had a lower inhibition effect. *E. coli* adhesion was 23.62% in the case of pre-treatment, 23.10% in the co-treatment assay, and 50.09% for the post-treatment.

**Figure 11:**
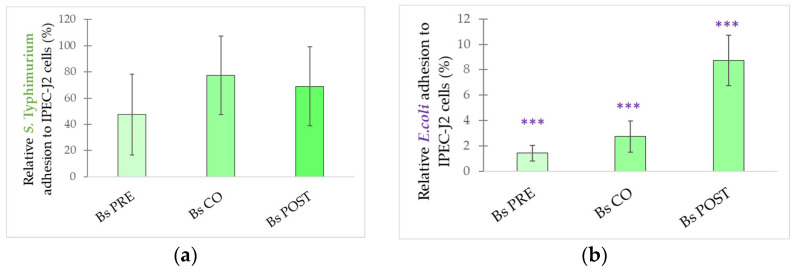
Inhibitory effect of *B. subtilis* on *S.* Typhimurium (**a**) and *E. coli* (**b**) adhesion to IPEC-J2 cells. *S.* Typhimurium and *E. coli* adhesion inhibitions were determined upon incubation with *B. subtilis* added 1 h before (pre-treatment), at the same time as (co-treatment), and 1 h after (post-treatment) the addition of *S.* Typhimurium and *E. coli*, respectively. *B. subtilis* was added in 10^8^ CFU/mL. **Bs PRE**: pre-treatment with *B. subtilis* 10^8^ CFU/mL + *E. coli* or *S.* Typhimurium 10^6^ CFU/mL; **Bs CO**: co-treatment with *B. subtilis* 10^8^ CFU/mL + *E. coli* or *S.* Typhimurium 10^6^ CFU/mL; **Bs POST**: post-treatment with *B. subtilis* 10^8^ CFU/mL + *E. coli* or *S.* Typhimurium 10^6^ CFU/mL. Data are shown as means with standard deviations and expressed as relative adhesion, considering the mean value of only *S.* Typhimurium or *E. coli* treated cells as 100%. n = 6/group. Significant difference: *** *p* ≤ 0.0001, in purple: compared with treatment with *E. coli*.

All treatment combinations with *B. subtilis* could inhibit *E. coli* adhesion to IPEC-J2 cells. Pre-treatment with *B. subtilis* was the most effective, followed by co- and post-treatment. *E. coli* adhesion was 1.42% in the case of pre-treatment, 2.73% in the co-treatment assay, and 8.73% for the post-treatment. However, when IPEC-J2 cells were challenged by *S.* Typhimurium, none of the treatment combinations with *B. subtilis* were able to inhibit the adhesion of the pathogenic bacterium (Figure 11).

## 4. Discussion

A healthy gut has four prerequisites: (1) proper barrier function, (2) intestinal immune fitness, (3) oxidative stress homeostasis, and (4) microbiota balance [2,44]. Probiotics have been shown to exert beneficial effects on the above-mentioned preconditions; however, the effect of these probiotics is strain-dependent [14,15,17,19,20]. The effect of *Enterococcus faecium* HDRsEf1 on IPEC-J2 cells has proven to be beneficial in many fields: enterotoxigenic *E. coli*-induced barrier impairment could be counteracted, *E. coli* adhesions were inhibited, and in addition *E. coli*-induced IL-8 secretion was modulated [41]. Furthermore, *Enterococcus faecium* NCIMB 10415 could reduce *S.* Typhimurium- and *E. coli*-induced ROS production in porcine intestinal cell culture (IPEC-J2) [45]. However, *L. rhamnosus* attenuated enterotoxigenic *E. coli*-induced damage to the IPEC-J2 cell barrier [46] and *Lactobacillus amylovorus* DSM 16698 inhibited enterotoxigenic *E. coli* adhesion to porcine epithelial cells (IPEC-1) and protected membrane damage induced by *E. coli* [47].

The objective of our study was to evaluate in vitro the probiotic potential of two candidates, *B. subtilis* and *B. licheniformis*, against pathogen-induced damages. The effects on paracellular permeability, inflammatory response, ROS production, and adhesion inhibition were investigated. Our hypothesis was that *B. licheniformis* and *B. subtilis* might (1) improve epithelial integrity, (2) reduce the secretion of proinflammatory cytokines, (3) alleviate the amount of reactive oxygen species, and (4) inhibit the adhesion of pathogenic bacteria. Two economically important swine pathogens, inducers of a wide range of gastrointestinal diseases in pigs, *S.* Typhimurium and *E. coli*, were chosen to challenge IPEC-J2 cells, in vitro [3,14,48,49].

Intestinal permeability is a good marker to monitor epithelial barrier function. Pathogens can disrupt barrier integrity, which leads to increased gut permeability, occurrence of diarrhoea, and leaky gut syndrome [44]. Probiotics have been shown to enhance the intestinal barrier function [17]. In our experiments, *B. licheniformis* alone had no significant effect on paracellular permeability. Interestingly, *B. subtilis* alone increased the paracellular permeability. Our experimental results with *B. licheniformis* are in line with studies showing that the use of probiotics alone might either not affect the integrity of the epithelial barrier or enhance the barrier function [50,51,52,53,54,55]. Lactobacilli had no effect on the barrier integrity of polarized intestinal epithelia [51]. *Enterococcus faecium* per se had no effect on the barrier integrity of IPEC-J2 cells; however, on Caco-2 cells, barrier function was enhanced [50]. In the case of *B. subtilis* alone, the increased FD4 flux indicates that the barrier function has been changed. *Enterococcus faecium* alone also decreased barrier integrity from 8 h incubation onward in IPEC-J2 cells [50]. *E. coli* or *S.* Typhimurium were able to disrupt the integrity of the barrier, in line with previous findings [56]. In our experiments, neither *B. licheniformis* nor *B. subtilis* was able to counteract the increased FD4 flux elicited by *S.* Typhimurium or *E. coli*. Unexpectedly, in some treatment combinations, the FD4 flux was further increased. However, other in vitro studies have shown that probiotic bacteria could prevent the barrier disrupting effects of *E. coli* [51,57]. This inconsistency might be because of the fact that probiotic properties are strain-dependent. When the effect of different probiotic strains (*Lactobacillus delbrueckii* ssp. bulgaricus no. 3; *Lactobacillus casei* no. 9; *Lactobacillus gasseri* no. 10; *Lactobacillus rhamnosus* OLL2838) on TNF-α-induced barrier impairment was investigated, only one strain (*Lactobacillus rhamnosus* OLL2838) was effective in counteracting the disruption of the barrier [58].

Pathogen-induced inflammation activates the immune system and various cytokines are synthetized. In the absence of challenge, low concentrations of proinflammatory cytokines (TNF-a, IFN-g, IL-1, IL-4, IL-6, IL-8) are indicators of immune fitness [44,55,56,57,58,59,60,61,62,63,64]. Previous studies have shown that probiotic bacteria can alter the expression of cytokines in epithelial cells [61,62]. IL-8 is a chemoattractant cytokine that can be produced by a variety of tissue and blood cells, but one of its major functions is to attract and activate neutrophils to inflammatory regions. IL-6 is a proinflammatory cytokine and is a stimulator of acute-phase proteins [63,64,65]. In our experiments, when IPEC-J2 cells were exposed to *E. coli* or *S.* Typhimurium, both IL-6 and IL-8 synthesis were significantly increased, a result also demonstrated by many previous studies [40,48,59]. When the inflammatory response was elicited by *S.* Typhimurium, all treatment combinations (pre-, co-, and post-treatment) with *B. licheniformis* could counteract the increase in IL-6 secretion. *B. licheniformis* has also been shown to decrease elevated IL-6 levels in vivo [1,66]. However, applying *B. subtilis*, only the pre-treatment with the probiotic bacteria could abrogate the elevated IL-6 synthesis. Interestingly, increased IL-8 production induced by *S.* Typhimurium was significantly further increased by the post-treatment with *B. licheniformis*. Others found that *Salmonella*-induced IL-8 secretion was decreased by *Bacillus licheniformis* ATCC 10716 [19]. The treatment of IPEC-J2 cells with *B. licheniformis* alone significantly increased the IL-8 secretion compared with the control, while the treatment with *B. subtilis* alone raised the IL-6 synthesis. A commensal microbe-mediated response might be similar to a pathogen-mediated response and increased proinflammatory cytokine secretions were also observed in other studies [48]. It is not only LPS that can induce inflammatory response, other metabolites may be involved and gram-positive bacteria might also induce inflammation [67]. Our data suggest that the pre-, co-, and post-treatment with *B. licheniformis* or *B. subtilis* offered no protection effect against *E. coli*-induced IL-6 and IL-8 secretion. Unexpectedly, pre-treatment with *B. licheniformis* further increased the secretion of IL-8 synthesis induced by *E. coli*. Others, however, found that *E. coli*-induced IL-8 elevation was counteracted by probiotic bacteria [40,41]. Similar to the GI tract, certain probiotic bacteria are more prone to counteract pathogen-induced inflammation than others [67]. Furthermore, animal models demonstrated that different taxa of microorganisms in combination can enhance pathogenic effects [67].

The measurement of ROS is a marker to monitor oxidative stress. Under oxidative stress, ROS are produced that lead to damage of proteins, lipids, DNA, and tissues [44]. Pathogens establish oxidative stress conditions in the intestines [68]. In our experiments, *E. coli* and *S.* Typhimurium induced an intracellular ROS burst in IPEC-J2 cells that could be significantly reduced by pre-, co-, and post-treatments with both *B. licheniformis* and *B. subtilis*. Thus, *B. licheniformis* and *B. subtilis* show powerful antioxidant properties upon pathogen challenge. Our finding agrees with other studies, where a similar beneficial effects of the probiotic strain *L. plantarum* ZLP001 on ROS generation has been proven [68].

One of the most important and most extensively studied probiotic properties is the ability to inhibit pathogen adhesion [69,70]. Our findings support that *B. licheniformis* and *B. subtilis* can effectively inhibit *E. coli* adhesion. Furthermore, *B. licheniformis* can also inhibit *S.* Typhimurium adhesion, suggesting that the inhibition effect depends on the pathogen strain applied. Significant adhesion inhibition was observed in the case of pre-, co-, and post-treatment, which indicates that *B. licheniformis* and *B. subtilis* were effective in the exclusion, competition, and displacement of pathogens. The effective displacement further indicates that established pathogen colonization could be disrupted. Our findings are similar to other recent studies reporting the adhesion inhibition of pathogens by probiotics [25,30,71].

Taken together, the treatment of IPEC-J2 cells with *B. licheniformis and B. subtilis* has shown effective antioxidant and inhibition properties upon *S.* Typhimurium and *E. coli* challenge, and treatment combinations also proved potent anti-inflammatory effects. However, the effect of the tested probiotics on paracellular permeability offered no obvious benefits here. Therefore, we suggest the use of these strains in combination with other probiotic species as multi-strain or multispecies mixtures, so that the beneficial health effects of different probiotics can be complemented by each other and synergistic activities can be exerted.

## Data Availability

All data that support the above-detailed findings can be obtained from the corresponding author upon request.
